# *Larinus berti* sp. n. (Coleoptera, Curculionidae, Lixinae) from North Africa

**DOI:** 10.3897/zookeys.342.5754

**Published:** 2013-10-14

**Authors:** Levent Gültekin, Miguel A. Alonso-Zarazaga

**Affiliations:** 1 Atatürk University, Faculty of Agriculture, Department of Plant Protection, 25240 Erzurum, Turkey; 2 Depto. de Biodiversidad y Biología Evolutiva, Museo Nacional de Ciencias Naturales, José Gutiérrez Abascal, 2, E-28071 Madrid, Spain

**Keywords:** *Larinus*, *Cryphopus*, taxonomy, new species, Lixini, Curculionidae

## Abstract

A new species, *Larinus berti* **sp. n.** is described from Morocco and assigned to subgenus *Cryphopus* Petri, 1907 (Curculionidae: Lixinae; Lixini). Diagnostic characters of the new species are large size, elongate-ovate body, bisulcate sub-quadrangular rostrum, triangularly raised dorsum of rostrum, flat subgena and submentum, Y-shaped apodeme of sternite VIII of female and thin nodulus of spermatheca.

## Introduction

The weevil genus *Larinus* Dejean, 1821 is considered a beneficial group in the fight against invasive thistles of the tribe Cardueae (Asteraceae) (Ter-Minasian 1967; Zwölfer et al. 1971; Gültekin 2006; Gültekin et al. 2008). According to the world catalogue by Alonso-Zarazaga and Lyal (1999), this genus is divided into four subgenera: *Cryphopus* Petri, 1907, *Larinus*, *Larinomesius* Reitter, 1924 and *Phyllonomeus* Gistel, 1856. In a recently prepared catalogue by Gültekin and Fremuth (2013), *Larinus* approximately consists of 100 species in the Palaearctic Region, with its highest species richness in the Mediterranean. Five species are assigned to *Cryphopus* and their distribution is confined to the Western Mediterranean (Gültekin and Fremuth 2013). Its diagnostic characters are the expanded outer apical angle of the protibia and the unequal length of the tarsal claws. This paper deals with the description of a new species from this subgenus.

## Materials and methods

Measurements were taken using an ocular micrometer attached to a Leica MZ75 stereo microscope and are defined as follows: body length: from anterior margin of eye to posterior margin of elytra; rostrum length: from apex of rostrum to anterior margin of eye in side view; prothorax length: from anterior margin to the posterior margin of scutellar lobe along midline. For the morphological study, dry adults were placed in lukewarm clean water overnight and their genitalia were dissected. Parts with muscles and other tissues were stored in 10% KOH overnight, cleaned with distilled water and 70% ethanol. Genitalia were observed and photographed in glycerine under a stereo microscope, and kept in glycerine microvials or allowed to dry and glued onto cards under the pinned specimens from which they were dissected. Photographs were taken with a Leica DFC 420 digital camera attached to the stereo microscope using LeicaLAS software for montage. The digital images were then imported into Adobe Photoshop 8.0 and CorelDRAW X4 for labelling and plate composition.

The material examined is deposited in the following collections:

MNCN Museo Nacional de Ciencias Naturales, Madrid, Spain.

SMNH The Swedish Museum of Natural History, Stockholm, Sweden.

## Taxonomy

### Larinus (Cryphopus) berti

Gültekin & Alonso-Zarazaga sp. n.

http://zoobank.org/EECCA20D-0AE2-4878-8432-7ABAA9613811

http://species-id.net/wiki/Larinus_berti

#### Diagnosis.

**Figures 1–7.**
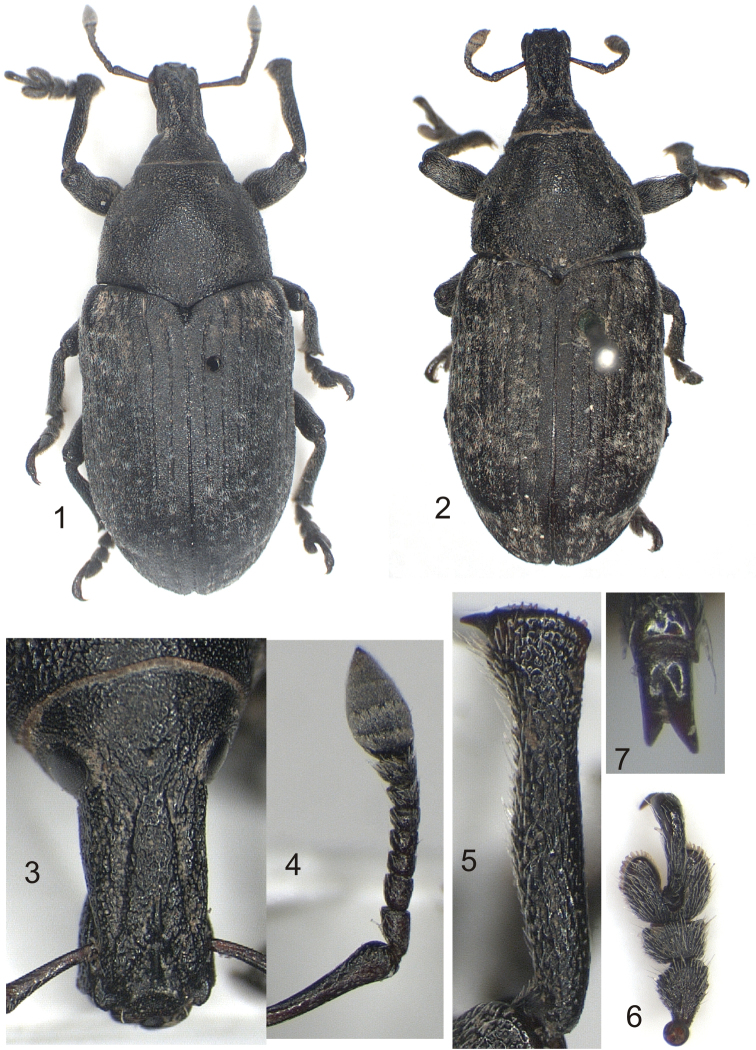
*Larinus berti* sp. n.: **1** holotypus (male) **2** paratypus (female) **3** rostrum (male) **4** antenna (male) **5** protibia (male) **6** protarsus (male) **7** claws.

*Larinus berti* Gültekin & Alonso-Zarazaga, sp. n. can be recognized because of its elongate-ovate large sized body (Figs 1–2), bisulcate sub-quadrangular rostrum, triangularly raised dorsum of rostrum (Fig. 3), flat subgena and submentum, Y-shaped apodeme of sternite VIII of female (Fig. 14) and thin nodulus of spermatheca. The new species is related to *Larinus griseus* Capiomont, 1874 but the latter clearly differs in the following characters: apodeme of sternite VIII of female is not Y-shaped, the subgena of rostrum is depressed, the submentum is distinctly raised at apex, and the central keel of dorsum is tricarinate.

#### Description.

*Measurements* (in mm): Body length: 13.60–14.40. Rostrum: length 2.70–2.80, width 1.50–1.60. Prothorax: length 4.00–4.20, width 5.30–5.50. Elytra: length 9.00–9.20, width 6.30–6.80.

*Body* elongate-ovate (Figs 1–2).

*Vestiture*. Ventral and lateral surface of head, dorsum of rostrum, pronotum and elytra with very short sparse greyish piliform scales; on elytra whitish grey pubescence forming small patches especially along striae; submentum, prosternum, medial part of metasternum, legs and abdominal ventrites with somewhat longer, denser and partly suberect hair-like pubescence; coxae, sides of metasternum and ventrite I, metanepisternum, mes- and metepimeron with bifid scales; mesosternum and mesanepisternum with 4- and 5-fid scales; scales on posterior part of metanepisternum and metepimeron very dense. Apical margin of prothorax with short dense piliform scales, longer on prosternum and ocular lobes. Tibial praemucro with a tuft of setae projecting towards uncus.

*Head* spherical, hind ventral margin with a small notch, vertex weakly visible, frons flat in female, slightly convex in male, frontal pit small, superficial, rounded. Eyes elliptical, weakly convex, ventral half narrower than dorsal. Rostrum (Fig. 3) sub-quadrangular in section, in dorsal view parallel-sided, weakly widened at apical third, with two deep longitudinal sulci reaching apical fourth and convergent caudad, area between sulci distinctly raised, with median keel at apical third, a transversely curved ridge present immediately before epistomal area, dorsolateral margins of rostrum obtuse, rostral pit invisible in female, distinct in male on median keel, minutely and coarsely punctured (as well as forehead); in side view straight. Scrobes with ventral margin partly visible dorsally. Antenna (Fig. 4) inserted about 0.30× from apex of rostrum in male, 0.40× in female. Scape slightly shorter than funicle, dorsoventrally depressed, weakly curved at middle, abruptly widened at apex, wider than desmomere 1, desmomeres 1–2 subconical, desmomere 1 about 1.30× as long as desmomere 2, desmomere 3 short, subisodiametric, about 0.65× as long as desmomere 2, desmomeres 4-7 gradually widening, desmomere 7 widest; club moderately elongate with acute apex, about 1.70× as long as wide.

*Prothorax* in dorsal view sub-trapezoidal, base moderately and triangularly arched towards scutellum, lateral margins of prothorax gradually and gently converging from base to apical half, rather strongly rounded apicad of it and then abruptly constricted in a short collar at apical 1/6; anterior margin very gently emarginate on dorsal part, evenly curved towards slightly developed postocular lobes; prosternum with anterior margin moderately emarginate. Pronotal surface convex, with dense, minute punctures, somewhat larger punctures scattered sparsely and partly confluent on prescutellar area.

*Scutellum* small and not clearly visible.

*Elytra* subparallel-sided in basal 2/3, gradually and roundly narrowed towards apex, constricted before mid-length; humeral calli moderately developed, preapical prominences distinct and located at end of intervals 4-7; interstriae flat, subequal in width on disc, narrower caudad, about 5× as wide as a stria on disc, interstria 10 wider than others in basal third, interstria 11 sinuate, weakly curved towards metanepisternum; striae mostly formed by rounded and separate punctures, these partly confluent along basal part and on posterior declivity, stria 10 sinuate and deeply sulciform.

*Abdomen* with ventrites 3 and 4 in male, and 3 only in female medially depressed.

*Legs*. Femora edentate, medially swollen, narrower than rostrum. Outer margin of protibia slightly, inner margin distinctly sinuate, apical part dilated outwards, about twice as wide as base, apex weakly curved, inner margin in male obtusely serrate, in female 3-4 small denticles present at apical half, serrate in basal half, denticles closest to uncus slightly larger than others (Fig. 5). Inner margins of meso- and metatibia nearly straight, outer margin slightly sinuate, mesotibia with 2-3 denticles, metatibia serrate in both sexes. Uncus sharp, moderately sized, gradually smaller from pro- to metatibia. Apical comb of spines short on protibia (longer on meso- and metatibia), bases of spines partly connate. Tarsi (Fig. 6) wide, tarsomere 3 1.40× as wide as tarsomere 2, 1.25× as wide as long, solea complete. Onychium stout, curved, gradually widened from base to apex, 0.65× as long as total length of tarsomeres 1-3; claws connate at basal third, of unequal length, inner claw shorter than outer, moderately divergent in apical half (Fig. 7).

**Figures 8–16.**
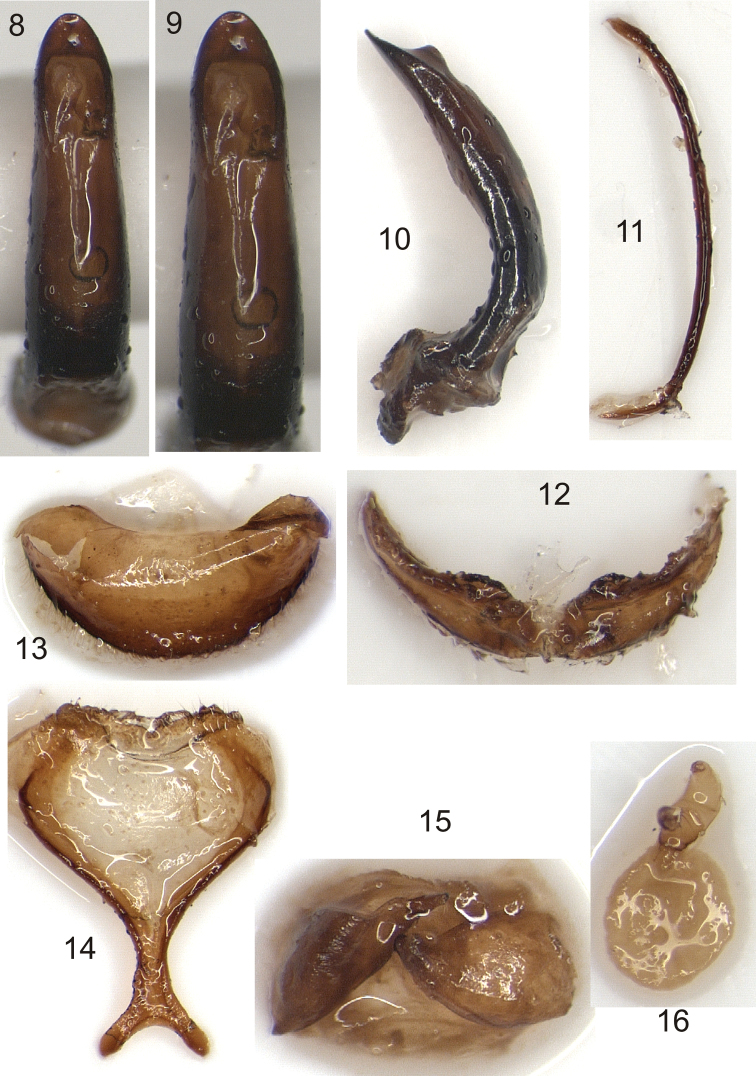
Terminalia and genitalia of *Larinus berti* sp. n. (**8–12** male; **13–16** female): **8–9** dorsal view of penis **10** lateral view of penis **11** spiculum gastrale **12** sternite VIII **13** tergite VIII **14** sternite VIII **15** coxite **16** spermatheca.

*Male terminalia and genitalia*. Penis in dorsal view stout, elongate, gradually narrowing from base to apical fourth, constricted in this part and narrowing again towards apex (Figs 8–9), incompletely sclerotized medially from basal third to ostium; apical plate triangular, 0.75× as long as wide; in lateral view, penis strongly curved at basal third, apical fourth almost straight (Fig. 10). Tegmen forming a ring with short ventral apodeme, parameroid lobes absent. Spiculum gastrale thin, stick-shaped, curved outwards, slightly shorter than penis (Fig. 11). Sternite VIII forming a single plate (Fig. 12), well sclerotized, apical margin with sparse, short setae.

*Female terminalia and genitalia*. Tergite VIII semicircular (Fig. 13), posterior margin with a dense row of hairs. Sternite VIII with lamina transversely oval, prolonged cephalad in an apodeme bifid at apex (Fig. 14); lateral arms wide, angularly arched outwards; margin angularly emarginate with a small medial triangular notch. Coxite well sclerotized, narrowed to apex, stylar base conical, stylus cylindrical, slightly longer than base (Fig. 15). Spermatheca nearly C-shaped, ramus well developed, distinctly wider than nodulus, the latter thin and cylindrical with a small tubercle at inner apical part, apex of cornu obtuse, gland well developed, subspherical (Fig. 16).

#### Variation.

Size variation is presented above under the Measurements section. A detailed variation cannot be presented because there are only two specimens available, and no further specimens have been traced in the rich Moroccan collections of the MNHN (Paris) and MNCN (Madrid). The female specimen shows a partly worn out vestiture, especially on elytra.

#### Type material.

Holotype (male) (Fig. 1), MOROCCO, Mischliffen, Md. Atlas, Marruecos, 2000 m, 5.VII.1988, Fdz-Rubio leg. [MNCN, Madrid]. Paratype (female) (Fig. 2), Afrique varia, “4”, [Chevrolat Coll.] [SMNH, Stockholm].

#### Etymology.

The new species is named after our good friend Bert Viklund (The Swedish Museum of Natural History, Stockholm).

### Key to the species of subgenus *Cryphopus*

The known species of the subgenus *Cryphopus* can be separated as follows:

**Table:** 

1	Body broadly ovate. Protibia strongly widened outwards, outer margin strongly sinuate. Claws strongly unequal in length	2
–	Body elongate-ovate. Protibia moderately widened outwards at apex, outer margin nearly straight. Claws moderately unequal in length	4
2	Length more than 15 mm. Apex of protibia hammer-shaped	*Larinus bombycinus* Lucas, 1847
–	Length less than 8 mm. Apex of protibia not hammer-shaped	3
3	Protibia with uncus and praemucro well separated	*Larinus ferrugatus* Gyllenhal, 1835
–	Protibia with uncus and praemucro tangent	*Larinus maroccanus* Capiomont, 1874
4	Subgena and apex of submentum flat. Apodeme of female sternite VIII bifid at apex	*Larinus berti* Gültekin & Alonso-Zarazaga, sp. n.
–	Subgena depressed, apex of submentum strongly raised. Apodeme of female sternite VIII simple	5
5	Rostrum in dorsal view subparallel-sided, thick, 1.25× as wide as maximum width of profemora, with a thin median keel	*Larinus reichei* Capiomont, 1874
–	Rostrum in dorsal view compressed at middle, ca. as wide as maximum width of profemora, with a thick median keel and two deep sulci	*Larinus griseus* Capiomont, 1874

## Supplementary Material

XML Treatment for Larinus (Cryphopus) berti
